# Clinical Manifestations of Hemorrhagic Fever with Renal Syndrome, Various Nosologic Forms and Issues of Hantavirus Infections Terminology

**DOI:** 10.3390/v17040578

**Published:** 2025-04-17

**Authors:** Evgeniy Tkachenko, Tamara Dzagurova, Guzel Galieva, Victoria Ivanis, Svetlana Kurashova, Petr Tkachenko, Alexandra Balkina, Dmitriy Trankvilevsky, Aydar Ishmukhametov

**Affiliations:** 1 Chumakov Federal Scientific Center for Research and Development of Immune-and-Biological Products of Russian Academy of Sciences, Institute of Poliomyelitis, Moscow 108819, Russiabalkina_as@chumakovs.su (A.B.);; 2 Department of Faculty Therapy, Bashkir State Medical University, Ufa 450008, Russia; gmukhetdinova@yandex.ru; 3 Department of Infectious Diseases, Pacific State Medical University (PSMU), Vladivostok 690990, Russia; 4 Department of Internal Disease Propaedeutics, Sechenov First Moscow State Medical University, Moscow 119991, Russia; 5 Department of Epidemiological Surveillance, Federal Center of Hygiene and Epidemiology, Moscow 117105, Russia

**Keywords:** hemorrhagic fever with renal syndrome, hantavirus pulmonary syndrome, hantaviruses, pathogen, clinical characteristics, clinical manifestations, epidemiological analysis, natural host, hot spot infection, nosological entity

## Abstract

Hemorrhagic fever with renal syndrome (HFRS) is the result of acute, zoonotic, natural foci hantavirus infections. It has serious social and medical importance due to its widespread distribution and the disease’s severity. There is a lack of effective etiotropic therapy and specific prophylaxis available. The aim of this review is to observe the etiological, clinical, and epidemiological features of nosologic HFRS forms in Russia, as well as differences and similarities with hantavirus pulmonary syndrome (HPS). The various clinical HFRS manifestations characterized diseases associated with Puumala, Kurkino, and Sochi hantaviruses in the Russian European part, and with Hantaan, Amur, and Seoul hantaviruses in the Russian Far East. Differences were observed for HFRS foci types based on biological characteristics and natural host population dynamics. As a result of clinical and epidemiological analysis six nosological forms were established, all of which were classified as “hemorrhagic fever with renal syndrome” according to the WHO’s expert recommendation from 1983 year. The study showed comparable taxonomic characteristics and determined the mechanism of human infection course for HFRS and HPS. The accumulated knowledge of this study allows for the combination of HFRS and HPS names into a common logical disease name “Hantavirus fever”.

## 1. Introduction

The discovery of hantaviruses, the causative agents of hemorrhagic fever with renal syndrome (HFRS) [1] and hantavirus pulmonary syndrome (HPS) [2], has stimulated active research worldwide, including Russia. Different scientific groups tried to isolate and identify distinct hantaviruses species (representatives of the genus *Orthohantavirus* according to strict compliance with the criteria of the International Committee on Taxonomy of Viruses) [3].

More than 100 hantavirus strains have been isolated from small mammals in the Russian territory. There have been hantaviruses with different immunologic and genetic properties. However, special attention has been placed on pathogen hantaviruses: Puumala, Kurkino, Sochi, Hantaan, Amur, and Seoul [4,5,6,7,8,9,10,11,12,13]. Diseases caused by these hantaviruses were distinguished and referred to as follows: HFRS-Puumala, HFRS-Kurkino, HFRS-Sochi, HFRS-Hantaan, HFRS-Amur, and HFRS-Seoul.

The etiological factors have two features for all six pathogenic HFRS types. The first is prolonged hantavirus infection persistence in the external environment. This feature promotes human infection without direct contact with the virus’s natural hosts [14]. The second is the direct hantavirus damage of the endothelial cells blood vessels (capillaries), which determines the disease course and outcome [15].

A total of 169,675 HFRS cases have been reported in 70 Russian subjects [16], including 167,062 cases in the European part (98.5% of the total HFRS cases number in Russia) and 2617 cases (1.5%) in the Asian part, mainly in the Far East territories among residents of the Primorsky, Khabarovsk, Jewish, and the Amur regions from 2000 to 2023.

**Figure 1:**
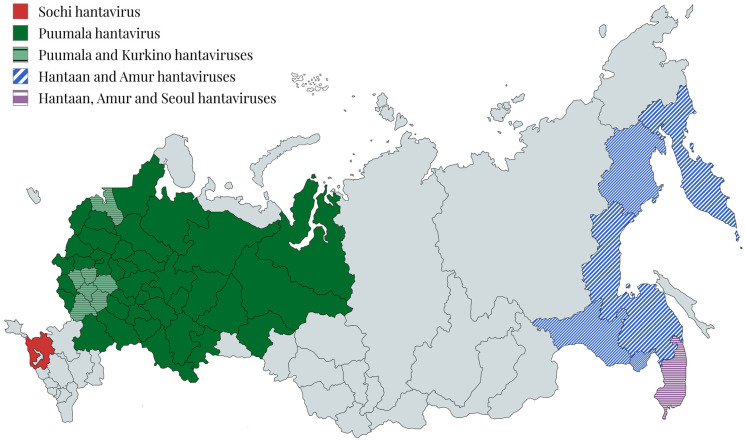
Geographic distribution of pathogenic hantaviruses in Russia.

The HFRS morbidity epidemiologic analysis revealed that approximately 98% HFRS cases were etiologically caused by Puumala hantavirus and only 2% by Hantaan, Amur, Seoul, Kurkino, and Sochi hantaviruses in Russia. These data indicate the leading etiological factor as Puumala hantavirus (Figure 1) [17].

## 2. Natural Foci of HFRS Etiological Forms in Russia

The natural foci of pathogenic hantaviruses and etiologically different HFRS clinical forms were distributed in the Russian European part and the Russian Far East (Figure 1).

Either the hantavirus species names or the rodent species names are used for the classification of the natural foci as the hantaviruses natural hosts [4].

The hot spot infection associated with the Puumala hantavirus, which we signify as “Puumala” foci, are distributed between the territories of more than 40 Russian European part subjects. The most active Puumala foci are located in the 14 regions territory in the broadleaved forests and coniferous forests of the Ural and the Middle Volga regions [17]. The Puumala hantavirus natural host and the human infection source is the bank vole, *Clethrionomys glareolus* [4]. The bank vole is a forest rodent that is rarely found outside the forest habitats, so it has little tendency to be synanthropic. Furthermore, in the foci of “Puumala” people themselves invade the natural habitat of the rodent’s territory (suburban areas near the forests). At the same time, the vole mass proliferation promotes animals to migrate into populated areas, especially in winter. This phenomenon occurs during thaws and creates additional conditions for human infection.

“Kurkino” natural foci are associated with the Kurkino hantavirus. They are common in the forest-steppe zone of the Russian central regions. The Kurkino hantavirus natural host as well as the human infection source is the western subspecies of the field mouse, *Apodemus agrarius agrarius* [17]. The field mouse populations are distributed in meadows and near water bodies during the warm season. These rodents inhabit haystacks, outbuildings, and household spaces in the autumn and winter. When the field mouse population reaches high local densities, the mice themselves “bring home” the infection to humans in epizootic cases in Kurkino foci. It should be emphasized that infection occurs both in rural areas and at the city outskirts. Small mammals have been captured in these areas in the epizootic years. The vast majority caught were field mice and the infection rate reached 90% among them [17].

The hot spot infection associated with Sochi hantavirus is distributed in the Krasnodar region subtropical zone [17]. The Sochi hantavirus natural host and the human infection source is the Caucasian wood mouse, *Apodemus ponticus.* The habitat area is limited for the Caucasian wood mouse by the zones of broad-leaved forests (including the lower belts of mountains) and floodplain oak forests of mixed forests. The number of Caucasian forest mice decreases from summer to fall and increases from fall to the next summer beginning in the Black Sea region. The other wood rodent populations increase from spring to fall and decrease from fall to spring. The feature mentioned for the Caucasian wood mouse increases the frequency of HFRS-Sochi outbreaks over the years. The highest HFRS-Sochi incidence rates are registered in the cities and districts of the Krasnodar region, which belongs to the foothill, mountain, and Black Sea zones.

Natural “Hantaan” foci associated with the Hantaan hantavirus are distributed in the Far Eastern Russian regions: Primorsky, Khabarovsky, Jewish, and Amur. These territories belong to zones of coniferous and deciduous forests, as well as to floodplain and meadow areas. The hantavirus natural host and the human infection source is the eastern subspecies of the field mouse, *Apodemus agrarius mantchuricus*. The field mouse is an ecologically plastic species that inhabits various biotopes: forest edges, shrub thickets, meadows, and wetlands. The most favorable habitat conditions for the field mouse are meadows, bushes, and especially agricultural areas. The field mouse is the dominant species, so you may find it everywhere. During the cold season, field mice often migrate into agricultural buildings (granaries, vegetable stores, etc.), creating additional infection risks for the rural population [18,19,20].

Natural foci of “Amur” are associated with the Amur hantavirus infection and are widespread in the Russian Far Eastern region [21]. The Amur hantavirus natural host and the source of human infection is the East Asian mouse, *Apodemus peninsulae*. The habitats of the East Asian mouse are cedar forests, oak forests, and shrub thickets. The Amur virus activity depends on the population’s size of the East Asian mouse, which is accompanied by a stable feed base (harvest of pine seeds, acorns, hazelnuts, etc.).

Natural “Seoul” foci are associated with Seoul hantavirus and are widespread in the southern Far East, where the gray rat (*Rattus norvegicus*) is the Seoul hantavirus natural host and the human infection source. Gray rats inhabit outbuildings, buildings in cities, and other populated areas, leading to increased contact with humans and favoring the HFRS-Seoul occurrence. There are difficulties in detecting HFRS-Seoul cases due to the mild clinical disease course. Patients do not seek medical help or receive another clinical diagnosis as a result [8,9,10,11].

It is important to point out, that the infected rodent population’s reproductive status plays a decisive role for the epizootic animal involvement in the natural host areas. Outside the breeding season, individuals from local settlements with increased rodent densities have the greatest chance to become infected with hantaviruses [22]. The epizootic situation of becoming worse; for example, in the optimal distribution zone of Puumala foci in spring and early summer. The proportion of sexually mature individuals increases sharply as a bank vole (*Clethrionomys glareolus*) reproduction results, which occurs every 2–4 years [23]. The same trend is observed for the Amur hot spot infection in the Primorsky region, where the East Asian mouse (*Apodemus peninsulae*) is the main host [24]. The western (*Apodemus agrarius agrarius*) and eastern (*Apodemus agrarius mantchuricus*) field mice population subspecies are epizootic active with an increase in local rodent density in the fall–winter period (foci “Kurkino” and “Hantaan” respectively) [23,24,25]. This phenomenon is accompanied by the synanthropic tendency and formation of density aggregates by the field mice during the cold season [26]. When the natural host habitats overlap, the different HFRS causative agents may coexist in the same area, which include the Kurkino and Puumala viruses in the Russian European part, and Hantaan and Amur viruses in the Russian Far East. Such foci function quite autonomously and often have asynchronous dynamics [22].

## 3. Clinical and Epidemiological Characteristics of HFRS Etiologic Forms

The analysis of the clinical and epidemiological characteristics revealed a number of fundamental differences among HFRS-Puumala, HFRS-Kurkino, and HFRS-Sochi in the Russian European region and HFRS-Hantaan, HFRS-Amur, and HFRS-Seoul in the Russian Far Eastern region.

The HFRS-Puumala incidence is characterized by a gradual increase from April to November and a subsequent decline until the end of January. In contrast, the HFRS-Kurkino incidence was registered between November and March, with the highest incidence in December and January. Most HFRS-Sochi cases detected in October–November are in the districts of Sochi and are comparable to the entire Krasnodar region [27]. HFRS-Puumala infection often occurs during short-term visits to the forest: during walks, fishing, hunting, when working in gardens, and living in country houses. HFRS-Kurkino infection occurs mainly during the animal’s care at their residence place or work. These include the disassembly of haystacks, transportation of hay, straw, and fodder, preparation and distribution of feed, and updating the straw litter in the stall. HFRS-Sochi patients in the vast majority of cases live in rural areas or in private construction houses near cities [27]. It was not possible to establish a link between the HFRS-Sochi occurrence and occupational patient activities or to determine the conditions for human infection by rodents, although it was reported the presence of rodents or their traces in more than 40% of the surveyed patients in households, outbuildings, and production facilities. The proportion of rural residents in the total number of patients with HFRS-Puumala was about 30%, while the proportion of patients with HFRS-Kurkino was 90% [27].

There are two epidemiological foci types. The first is rural, where the cases of HFRS-Hantaan and HFRS-Amur predominate. The second is urban, where the cases of HFRS-Seoul are the most often registered in the Far Eastern Russian region [28]. Two seasonal increases registered for morbidity in rural foci: spring–summer (May–July) and fall–winter (October–November) with a peak in November. The highest number of HFRS-Seoul cases was revealed from February to May, with a peak in March in urban foci. The ratio of male-to-female cases in rural foci is 7:1 and in urban foci is 3:1. Infections of personnel are more often related to urban foci. Agricultural workers such as tractor drivers, mechanics, field and livestock farmers, and forestry workers are exposed to infection during rural outbreaks. The domestic cases in proportion are twice as high in urban as in rural foci. Infection with HFRS-Hantaan occurs due to short visits to the forest while fishing in rivers close to the forest, or while hunting. Practically the same is typical for HFRS-Amur infection (when picking berries, mushrooms, pine cones, and nuts) usually in late summer and fall [28]. HFRS-Seoul patients contact with gray rats both at the residence place and at the work place [9]. The HFRS-Seoul incidence belongs to the age group of 20–49 years. The vast majority of patients are male. It is known that the gray rat mainly infests and inhabits basements, garages, and utility and storage rooms [28,29]. Infected gray rats are present in almost all trapping areas in urban foci. This indicator reaches 90% for some places [9]. Obviously, the largest HFRS-Seoul patient number was found for urban foci among residents of the second floors, and electricians and plumbers working in basements.

Clinical signs are not specific for HFRS diagnosis during the first three days of illness. Taking into consideration the pathogenic mechanism of HFRS, in our previous studies we have identified the constant presence of triad symptoms, including fever, low platelet count proteinuria, and hematuria [30,31], in Russian. Any acute fever considered suspicious for HFRS in patients who live in the areas of HFRS natural foci or have visited the foci within one to one and a half months before the onset of the disease (the incubation period duration). With a typical disease course and experienced medical staff, the HFRS diagnosis does not pose any difficulties. The HFRS diagnosis should considered conclusive if the clinical signs are consistent with the characteristic disease course and the epidemiological history.

The HFRS disease’s clinical course has several manifestations, which are divided into mild, moderate, and severe forms. Mild hemodynamic and renal disorders are noted in the mild form, where body temperature does not rise higher than 38 °C, a moderate decrease in diuresis with normal blood urea, creatinine up to 130 μmol/L, normocytosis, low proteinuria, and hematuria. Toxic syndrome (fever, headache, dizziness, fainting, nausea, and anorexia) is more pronounced in the moderate form than in the mild form. Body temperature rises to 39.5 °C, and moderate hemorrhagic syndrome appears, other parameters are as follows: oliguria–300–900 mL per day, blood urea–8.5–19 mmol/L, creatinine 131–299 μmol/L, WBC 8.0–14.0 × 10^9^/L, proteinuria, and hematuria. Toxic syndrome significantly affects the patient’s condition, where their body temperature is above 39.5 °C, marked hemorrhagic syndrome, uremia, daily diuresis–200–300 mL, blood urea above 19 mmol/L, creatinine above 300 μmol/L, leukocytosis above 14.0 × 10^9^/L, heavy proteinuria, and hematuria. HFRS complications are bleeding, hemorrhage, infectious toxic shock syndrome, acute heart failure, pulmonary edema, uremic coma, renal eclampsia, rupture of the renal capsule, and secondary bacterial infections [32].

The differences identified for HFRS course are complex according to our long-term observations. We emphasize the importance of the following parameters: multisystem lesions, clinical manifestations, microcirculation disorders, and finally, the severity of central hemodynamics. They depend on the etiology of HFRS forms: HFRS-Puumala, HFRS-Kurkino, HFRS-Sochi, HFRS-Hantaan, HFRS-Amur, and HFRS-Seoul [4,28,32,33,34,35,36,37,38,39].

HFRS-Puumala is the most common etiologic form of the disease in Russia. About a quarter of patients with HFRS-Puumala have a mild form, half of the patients have a moderate form, and another quarter have a severe form [4]. Hemorrhagic syndrome occurs in a quarter of patients. According to the laboratory test results, a decrease in the urine specific gravity is significant. The mortality rate in patients with HFRS-Puumala is 0.4–1%.

HFRS-Kurkino disease usually proceeds in a mild form, and severe forms are observed for the clinical infection course in a quarter of patients. Hemorrhagic appearance is rarely observed. The HFRS-Kurkino clinical course peculiarities include visual disturbances, hyperemia of the face, and oropharynx. Laboratory abnormalities characterized by lymphopenia and a shift in the leukocyte formula to the left with plasma cells are rarely detected, and a greater increase in erythrocyte sedimentation rate and a lesser decrease in the urine specific gravity are observed. The mortality rate in patients with HFRS-Kurkino does not exceed 0.5% [36].

HFRS-Sochi is the most severe HFRS form among the etiological forms of the disease registered to date. More than half of the patients with HFRS-Sochi have a severe form with significant hemorrhagic manifestations. The majority of patients with HFRS-Sochi have gastrointestinal disorders in the form of abdominal pain, nausea, vomiting, and diarrhea. Every tenth patient shows liver damage signs, including increased bilirubin and transaminase levels. A high mortality rate is marked as up to 14% [36].

The HFRS-Hantaan disease is characterized by considerable clinical severe, cyclic, and periodic features of toxic syndrome. At the same time, homeostasis disorders increase (elevated WBC, neutrophilosis, lymphopenia, and thrombocytopenia). As a rule, as the hemorrhagic syndrome progresses, skin rashes (from linear petechiae to ecchymoses) and bleeding (nasal, genital, renal, and gastrointestinal) occur. In this case, the syndrome of hemodynamic (central and microcirculatory) disorders is moderately pronounced, in which rare occurrences of toxic shock, hypotension, and neurological disorders are observed. The patient’s appearance characteristics include swelling and hyperemia of the skin on the face, neck, chest, and injection of scleral vessels. Kidney damage (acute renal failure syndrome (ARF), period of oliguria, and anuria) is the most characteristic HFRS-Hantaan sign and includes repeated vomiting, which sometimes takes on the character of indomitable, constant dull pain in the abdominal and lumbar region, thirst, hiccups, oliguria, anuria, face edema, shins, and exudative effusions in the cavities (hydrothorax, ascites, hydropericardium, parametritis). During this period there is persistent hypertension and an increase in blood nitrogen (increased urea and creatinine levels), which can last up to 5–9 days. The mortality rate in patients with HFRS-Hantaan is 5–10%.

HFRS-Amur is characterized by a more pronounced acyclic course, the toxic syndrome severity, central hemodynamic disorders signs, the frequency of toxic shock, and electro-cardiography (ECG) changes [39]. ARF syndrome is one of the most HFRS-Amur characteristic manifestations. The clinical signs of ARF appear already in the disease’s first days against the background of hyperthermia. The oliguria and anuria period occurs rapidly, and the creatinine and urea levels increase by 5–10 times. The hemorrhagic syndrome is less typical for HFRS-Amur and manifests itself only in the form of a single petechiae and hematuria. Respiratory syndrome and hepatitis syndrome are rare. The mortality rate in patients with HFRS-Amur is 5–10% [39].

The HFRS-Seoul mainly occurs in mild to moderate severity forms. In the disease’s initial phase, the symptoms of acute respiratory disease usually predominate in patients. Against the background of weakly expressed and rapidly disappearing signs of renal pathology (the duration of polyuria does not exceed 3–4 days), most patients have hepatosplenomegaly, jaundice, hyperbilirubinemia, elevated alanine aminotransferase (ALT), and aspartate aminotransferase (AST). An HFRS-Seoul form peculiarity is frequent liver damage. An increase in the serum bilirubin concentration is found for one among five patients, and an increase in ALT and AST activity—in more than half of patients. Lethal outcomes are not observed for HFRS-Seoul practically [39].

As a result of clinical and epidemiological characteristics analysis of HFRS caused by different hantaviruses (Puumala, Kurkino, Sochi, Hantaan, and Amur, Seoul), the existence of six different hantavirus disease entity forms under one name “hemorrhagic fever with renal syndrome” (ICD-10, heading A98.5) established in Russia [40].

## 4. Terminology Question

The name “hemorrhagic fever with renal syndrome” was proposed by Chumakov M.P. in 1954 [41]. In 1983, WHO experts officially recommended this name [42] in order to unify the etiological and clinical similar diseases designation and to avoid the use of numerous names by different authors in different infection study periods (in the USSR: infectious nephroso-nephritis, Churilov’s disease, hemorrhagic nephroso-nephritis, Yaroslavl, Kalinin, Ural, Tula fevers, Transcarpathian hemorrhagic fever; worldwide: Scandinavian epidemic nephropathy, epidemic nephropathy, Songo fever, epidemic hemorrhagic fever, Korean hemorrhagic fever, and epidemic glomerulonephritis) [43,44,45,46,47,48,49,50].

A comprehensive disease name analysis was carried out and described in detail by the well-known Russian professor Boris Zalmanovich Sirotinin (1928–2019) in 1994 [51].

The name HFRS and synonyms analysis demonstrate that one or another external disease feature is dominant as a term, which is far from its main characteristics [51]. One term emphasizes the epidemic disease nature, the presence of hemorrhagic syndrome, and fever (epidemic hemorrhagic fever). The other term includes the epidemic nature of kidney damage (epidemic nephropathy). The third term variation considers the geographic feature, the hemorrhagic syndrome, and fever (Korean hemorrhagic fever). Finally, the term HFRS proposed by the WHO experts also reflects the external clinical manifestations of the disease. Thus, the accepted name lacks etiologic, pathogenetic, or morphologic characteristics.

The majority of disease names reflect the presence of hemorrhagic manifestations. Indeed, the hemorrhagic syndrome in the form of small hemorrhages, more often subcutaneous hemorrhages, and even fewer various internal hemorrhages, occupies an important place in the clinic signs. However, there are disease types without hemorrhagic manifestations in the mild forms. Sometimes hemorrhagic manifestations are absent even in severe disease forms. Hemorrhages are less significant in the clinical course of epidemic nephropathy. The characteristic was not included in the name of this disease type. Obviously, this variant of the disease is not suitable for classification as HFRS. At the same time, it is possible that HFRS does not include this feature in the name of the disease. On the other hand, hemorrhagic manifestations are observed in a large number of different infectious and non-infectious diseases. They include other types of hemorrhagic fevers, which at the same time differ in etiological, pathogenetic, and clinical characteristics. Thus, hemorrhagic syndrome is not an exclusive HFRS feature, especially depending on the other manifestations.

Another HFRS feature is a fever, which also appears in various other disease names. It is impossible to overlook all word combinations with the term “fever”, the same situation is referred to as the term hemorrhages. On the other hand, a pronounced hemorrhagic syndrome usually occurs against the background of an already normal temperature. However, fever is indeed an integral disease part. It occurs even before the first hemorrhagic sign appears as a rash on the skin, enanthema of the palate, and, above all, well-defined hemorrhages. As a rule, the fever disappears after 1–1.5 weeks of the disease and the temperature normalizes. At this point, the disease acquires the most distinctive and specific features in the form of ARF development [51]. It is possible that the kidney’s involvement in the pathological process, which reaches its climax at this time, causes the disappearance of the fever. Various inflammatory processes proceed feverishly against a background of acute and chronic renal failure. However, the fever in this disease is short-lived and has no characteristic signs. The only HFRS fever property is that the temperature normalization is accompanied usually by a deterioration in the patient’s condition [51]. Therefore, neither hemorrhagic manifestations nor fever are characteristic signs of the disease, and cannot be useful for its clinical recognition. When the clinical picture is devoid of renal changes in the presence of hemorrhagic diathesis and fever, such cases pose great difficulties for clinical diagnosis, even in epidemic outbreaks in endemic areas. They are a source of various diagnostic errors if serologic diagnostic methods are not used to detect the specific antibodies present against hantaviruses, the causative HFRS agents [52].

The most important disease sign is the renal syndrome. Renal manifestations are mentioned only in two disease names (epidemic nephropathy and HFRS). The term “renal syndrome” reveals the essence of the disease and the most important clinical characteristic. This term gives this disease the specificity that distinguishes it from the numerous other hemorrhagic fevers in which the renal pathology is completely absent and not marked as HFRS. Finally, it is important to mention that despite the damage to many organs and systems during HFRS, survivors may find disorders primarily in the kidneys after the disease. Thus, the kidneys are the epicenter of internal organ pathology in patients and HFRS survivors [51]. The term “renal syndrome” occupies the last position in the name of the disease, while it is this clinical manifestation that refers to the nature of renal pathology. However, it does not determine exactly whether it is a glomerular, interstitial, or tubulointerstitial disorder. The renal pathology occupies a central place among the other organs and systems involved in the pathologic HFRS pathway. This pathology is classified as viral acute tubulointerstitial nephritis. The main pathogenic features of this disease are vascular damage and the subsequent appearance of hemorrhages. That is not typical for the etiology of other forms of tubulointerstitial nephritis. Thus, we have identified the following features of HFRS: etiological, pathogenetic, and morphological.

This consideration led to the proposal to call this disease “acute hantaviral hemorrhagic tubulointerstitial nephritis” [51]. This corresponds to the modern approach to the diseases nosology. The name includes the necessary classification features: etiology, pathogenesis, morphology, and the most important clinical feature. The name of the disease receives a certain nosological position in the renal pathology structure. In addition, the name “HFRS” includes only the external features of the disease, which are not always present in the clinical manifestations.

At the same time, the HFRS disease nature is not limited by renal syndrome, as the lungs are also target organs in HFRS due to the respiratory infection mechanism. The respiratory system damage characterizes not only HPS but also HFRS [37,53,54,55]. There are HPS cases described as caused by hantaviruses etiological factors, the causative HFRS agents [56,57]. Conversely, renal failure occurs with HPS [58]. Therefore, the lesion of the respiratory system during the hantavirus infection course, regardless of the pathogen type, has a primary determined character. Its clinical signs are expressed by minor changes in the lungs or the development of a severe course of HPS [59]. The human infection HPS mechanism is identical to those of HFRS. They are linked by clinical manifestations diversity, the epidemiologic and epizootic processes, and, finally, the taxonomic characterization of the viruses [60,61,62]. Because of this similarity, researchers suggest that HFRS and HPS should be combined into a common logical name for the disease “Hantavirus infection” [63,64,65] or “Hantavirus fever” [66,67]. In our opinion, the most consistent name for inclusion in ICD-11 is “Hantavirus fever”, similar to the names of diseases such as Dengue fever, Yellow fever, West Nile fever, and Mosquito fever.

Etiological differences between the HFRS and HPS clinical entity have no bearing on the principles of their treatment. They are remaining pathogenic and symptomatic nature for all forms at this time.

## 5. Conclusions

The relationships between hantaviruses and their warm-blooded hosts and the pathogen transmission mechanisms within the binomial parasitic system “virus–warm-blooded host” determine the specific spread, local area, and hot spot infection functioning.

The natural foci of human pathogen hantaviruses and the etiologically different clinical HFRS forms caused by them include locations in the Russian European part with the hantaviruses Puumala, Kurkino, and Sochi, and with the hantaviruses Hantaan, Amur, and Seoul in the Russian Far East.

The main differences are formed for HFRS types due to the biology and population dynamics peculiarities of the pathogenic to humans hantaviruses natural hosts–the bank vole *Clethrionomys glareolus*, western subspecies of the field mouse, *Apodemus agrarius agrarius*, Caucasian wood mouse, *Apodemus ponticus*, eastern subspecies of field mouse, *Apodemus agrarius mantchuricus*, East Asian mouse, *Apodemus peninsulae*, and gray rat, *Rattus norvegicus*. The epizootic and epidemic processes associated with the species natural hosts characteristics appears to be particularly close due to the transmission respiratory route and the hantavirus species specificity. The carrier rodent biology determines in particular the contact type of the population in the focus area with them.

As a result of the clinical and epidemiological analysis caused by the immunologically and genetically distinct hantaviruses Puumala, Kurkino, Sochi, Hantaan, Amur, and Seoul, six independent disease entities of hantavirus infection have been identified, which exist under the name “hemorrhagic fever with renal syndrome”.

Due to the similarity of clinical signs, epidemiological and epizootic processes, taxonomic virus characteristics, and infection route in humans, we propose to combine the names HFRS and HPS into a common logical name for the disease “hantavirus fever”.

## Abbreviations

The following abbreviations are used in this manuscript:

HFRS	Hemorrhagic fever with renal syndrome
HPS	Hantavirus pulmonary syndrome
ARF	Acute renal failure syndrome
ECG	Electro-cardiography
ALT	Alanine aminotransferase
AST	Aspartate aminotransferase
